
JOURNAL INFORMATION
==============================
NLM Title Abbreviation: Exp Physiol
Journal ID: Exp Physiol
NLM Journal ID: 9002940
Journal ID: EPH

Experimental Physiology

ISSN: 0958-0670
EISSN: 1469-445X
Publisher: Wiley

ARTICLE INFORMATION
==============================
PMCID: PMC13422960
DOI: 10.1113/eph.70407
Article ID: EPH70407
Article version: 1
Subjects: Issue Information, Issue Information

Issue Information

Electronic publication date: 2026 Jul 30
Publication date: 2026 Aug
Volume: 111
Issue: 8
First page: 3858
Last page: 3862
Copyright: © 2026 The Authors. Experimental Physiology published by John Wiley & Sons Ltd on behalf of The Physiological Society.
License: This is an open access article under the terms of the http://creativecommons.org/licenses/by/4.0/ License, which permits use, distribution and reproduction in any medium, provided the original work is properly cited.
License URL: https://creativecommons.org/licenses/by/4.0/

Figure count: 0
Table count: 0
Page count: 5

source-schema-version-number: 2.0
cover-date: 1 August 2026
details-of-publishers-convertor: Converter:WILEY_ML3GV2_TO_JATSPMC version:6.8.4 mode:remove_FC converted:30.07.2026

==============================

### Experimental Physiology

Open Access

Volume 111 | Issue 8 | 1 August 2026

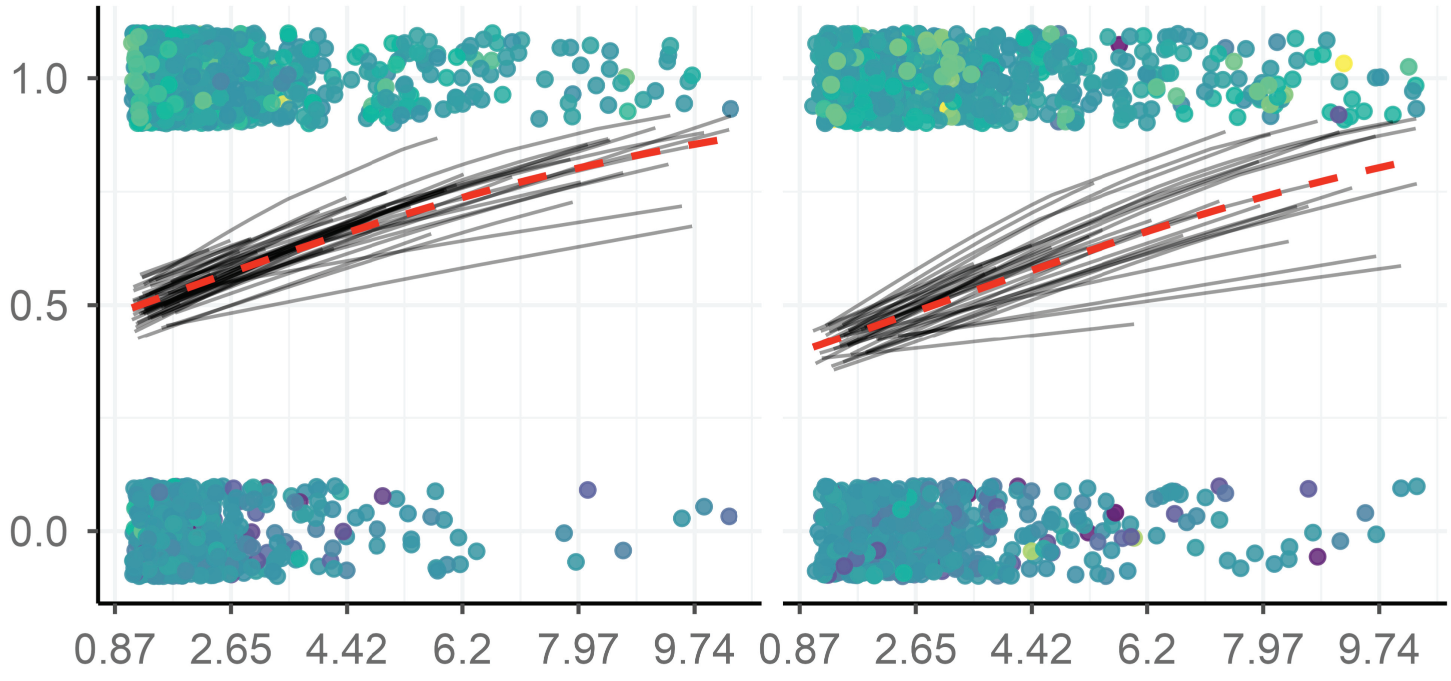

WILEY

The Physiological Society

150 YEARS
1876 2026
LEADING DISCOVERY SCIENCE

### Experimental Physiology

Open Access

Integration and Translation

Volume 111/Issue 8/
1 August 2026

### EDITORIAL BOARD

**EDITOR-IN-CHIEF**
Damian Bailey *Pontypridd, UK*

**DEPUTY EDITOR-IN-CHIEF EUROPE**
Ronan Berg *Copenhagen, Denmark*

**DEPUTY EDITOR-IN-CHIEF USA**
David Poole *Manhattan, KS, USA*

**SENIOR ETHICS EDITOR**
Susan Barman *East Lansing, MI, USA*

**STATISTICAL EDITOR**
Cody Durrer *Copenhagen, Denmark*

**SENIOR REVIEWS EDITOR**
Toby Mündel *Ontario, Canada*

**CONNECTIONS EDITOR**
Prem Kumar *Birmingham, UK*

**SENIOR EDITORS**
Dana Almohazey *Dammam, Saudi Arabia*
Jill Barnes *Madison, WI, USA*
R. Matthew Brothers *Arlington, TX, USA*
Kanokwan ‘Mandy’ Bunsawat *Iowa City, IA, USA*
Kirstene Callø *Copenhagen, Denmark*
Katrina Colafella *Victoria, Australia*
Ellen Dawson *Liverpool, UK*
Colleen Deane *Southampton, UK*
Peter Zu Eulenburg *Munich, Germany*
Federico Formenti *London, UK*
Julien Ochala *Copenhagen, Denmark*
Shigehiko Ogoh *Kawagoe, Japan*
Jason Peart *Gold Coast, Australia*
Peter Rasmussen *Copenhagen, Denmark*
Lee Romer *London, UK*
Harry B. Rossiter *Los Angeles, CA, USA*
Bill Sheel *Vancouver, Canada*
Mike Stembridge *Cardiff, UK*

**REVIEWING EDITORS**
Tatsuro Amano *Niigata, Japan*
Claire Badenhorst *Palmerston North, NZ*
Tracy Baynard *Boston, MA, USA*
Hannah Caldwell *Copenhagen, Denmark*
Havovi Chichger *Cambridge, UK*
Silvia Conde *Lisbon, Portugal*
Joseph Costello *Portsmouth, UK*
Jennifer Davies *Cardiff, UK*
Judy Delp *Tallahassee, FL, USA*
Aimee Drane *Cardiff, UK*
Esther Dupont-Versteegden *Lexington, KY, USA*
Andrea Fellet *Buenos Aires, Argentina*
Carrie Ferguson *Torrance, CA, USA*
Igor Fernandes *Niteroi, Brazil*
Ryan Hoiland *Kelowna, Canada*
Seth Holwerda *Kansas City, KS, USA*
Anna Hudson *Adelaide, Australia*
Matthew Kay *Washington, DC, USA*
James Lang *Ames, Iowa, USA*
Jacqueline Limberg *Columbia, MO, USA*
Fiona McBryde *Auckland, NZ*
Carmen De Miguel *Birmingham, AL, USA*
Niels Olesen *Copenhagen, Denmark*
Leah Reznikov *Gainesville, FL, USA*
Kleiton Silva *Camden, NJ, USA*
Sarah Short *Iowa City, IA, USA*
Nathaniel Szewczyk *Nottingham, UK*
Can Ozan Tan *Boston, MA, USA*
Mike Tymko *Vancouver, Canada*
Kai Yang *Guangzhou, China*
Song Yao *Melbourne, Australia*
Gensheng Zhang *Hangzhou, China*

**FUTURE LEADERS**
Edward Ashworth *Basel, Switzerland*
Sophie Broome *Melbourne, Australia*
Callum Brownstein *Newcastle, UK*
Daniel Craighead *Minneapolis, MN, USA*
Robert Lakin *Toronto, Canada*
Jedd Pratt *Manchester, UK*
Fabio Sarto *Padua, Italy*
Hollie Speer *Canberra, Australia*

**CONSULTANT EDITORS**
Richard Burden *Bisham, UK*
Nic Green *Bedfordshire, UK*
Julie Greeves *Salisbury, UK*
Denis Noble *Oxford, UK*
Nanduri Prabhakar *Chicago, IL, USA*

**PUBLISHER**
Alex Stewart (astewart@physoc.org)

**DEPUTY MANAGING EDITOR**
Hollie Evans (hevans@physoc.org)

**PEER REVIEW MANAGER**
Diana Jones (ephjournal@physoc.org)

**PEER REVIEW COORDINATOR**
Paroma Guha (ephjournal@physoc.org)

**EDITORIAL OFFICE**
The Physiological Society
Hodgkin Huxley House
30 Farringdon Lane, Clerkenwell
London EC1R 3AW

**PRODUCTION OFFICE**
K. Nandini (EPH@wiley.com)

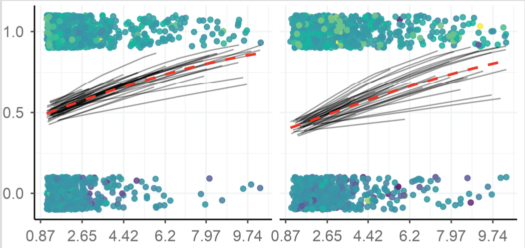

**Cover Illustration**
O’Sullivan et al., demonstrate that very low amplitude muscle activity significantly increases the likelihood of evoking a muscle response with transcranial magnetic stimulation (TMS). This demonstrates that even minimal muscle activation can bias ‘resting’ TMS measures of motor excitability. See O’Sullivan et al. Pages 3492–3501.

**Open Access and Copyright**
All articles published by Experimental Physiology [submitted after 18 October 2022] are fully open access: immediately freely available to read, download, and share. All articles [accepted from 18 October 2022] are published under the terms of a Creative Commons license.
Copyright on any research article published by Experimental Physiology is retained by the author(s).
Further information about open access licenses and copyright can be found at: https://authorservices.wiley.com/author-resources/Journal-Authors/licensing/licensing-info-faqs.html#4. Information on the licenses used by Experimental Physiology can be found at: https://physoc.onlinelibrary.wiley.com/journal/1469445X.

For submission instructions and all other information visit: https://physoc.onlinelibrary.wiley.com/journal/1469445X

The Physiological Society and The Physiological Society logo are trade marks belonging to The Physiological Society and are registered in the UK and in the EU Community, respectively.

*Experimental Physiology* publishes high quality, original, physiological research papers that give novel insights into fundamental homeostatic and adaptive responses in health and papers that further our knowledge of pathophysiological mechanisms in diseases.

Experimental Physiology
Open Access
Integration and Translation

### INFORMATION AND GUIDANCE FOR AUTHORS

**Full version online at http://ep.msubmit.net/cgi-bin/main.plex?form_type=display_requirements**

### ORIGIN

*The Quarterly Journal of Experimental Physiology*, founded in 1908 by Sir Edward Sharpey-Schafer, Professor of Physiology, University of Edinburgh, became a publication of The Physiological Society in 1981. The title was changed to *Experimental Physiology* in 1990.

### SCOPE

*Experimental Physiology* publishes research papers that report novel insights into homeostatic and adaptive responses in health, as well as those that further our understanding of pathophysiological mechanisms in disease. We encourage papers that embrace the journal's orientation of translation and integration, including studies of the adaptive responses to exercise, acute and chronic environmental stressors, growth and aging, and diseases where integrative homeostatic mechanisms play a key role in the response to and evolution of the disease process. Examples of such diseases include hypertension, heart failure, hypoxic lung disease, endocrine and neurological disorders. We are also keen to publish research that has a translational aspect or clinical application. Comparative physiology work that can be applied to aid the understanding human physiology is also encouraged.

Manuscripts that report the use of bioinformatic, genomic, molecular, proteomic and cellular techniques to provide novel insights into integrative physiological and pathophysiological mechanisms are welcomed.

We are also interested in methodological papers reporting important new developments in physiological techniques that could lead to significant new insights.

Papers which observe a significant new physiological behaviour or phenomenon which has the potential to alter the understanding of that field, but which have not yet identified the mechanisms may also be considered. Such papers should demonstrate the experimental examination and exclusion of a number of plausible mechanisms for the important novel phenomenon.

### Article Types

In addition to original research, *Experimental Physiology* consider the following article types for publication:
*Case studies, Short Communication, Connections, Reviews, Full Reviews, Hot Topic Reviews Myths and Methodologies and Symposium Reports.*
Review articles are normally by invitation but proposals may be submitted for consideration.
Commentary articles in the form of Viewpoints and Letters are also published.
For full guidelines on all published article see full guidelines.

C O P E COMMITTEE ON PUBLICATION ETHICS

This journal is a member of and subscribes to the principles of the Committee on Publication Ethics

### ETHICS OF EXPERIMENTATION

**Animal Experiments** - see full journal policy at https://physoc.onlinelibrary.wiley.com/hub/animal-experiments

Experimental Physiology endorses the ARRIVE Guidelines for reporting *in vivo* experiments: http://onlinelibrary.wiley.com/doi/10.1113/expphysiol.2010.053793/full

Authors of papers submitted to Experimental Physiology must adhere to the journals' principles and standards for reporting animal experiments.

Authors should read these guidelines to ensure that their research and experiments adhere to our exacting standards.

Euthanasia/humane killing: Animals must be killed using methods approved for that species, stage of development and size. The standards required by the journals are those of the UK/European Union legislation, set out in in ASPA Schedule 1 in the UK and in Annex IV in the European Directive 2010/63/EU. Methods not on Schedule 1 or Annex IV could be permissible but even where the method used conforms with national guidelines, the onus is on the author to provide scientific evidence to demonstrate that it is at least as humane as those on Schedule 1 and Annex IV.

Our guidelines have been outlined in the Editorial 'Principles and standards for reporting animal experiments in Experimental Physiology'.

**Confirmation of compliance**

Authors must state in the Methods section that the investigators understand the ethical principles under which the journal operates and that their work complies with the animal ethics checklist as outlined in the Editorial.

**Human Experiments** - See full journal policy at https://physoc.onlinelibrary.wiley.com/hub/human-experiments

The Editorial Boards are concerned that all work published in The Journal of Physiology and Experimental Physiology should have been conducted according to the highest standards of safety and ethics. Legislation and accepted practice concerning human experimentation vary from country to country and consequently it is difficult to define absolute requirements. However, work with human subjects should conform to the standards set by the Declaration of Helsinki (last modified in 2013), the Medical Research Council's online guidance on the use of human tissue, and the guidelines set out below. The objectives must be to ensure that all risks are minimized and that subjects are not injured and do not feel they have been abused as a result of participating in the study. Any definition of abuse will include excessive or unexpected pain or discomfort experienced during the experiment. Note, in the case of experiments involving minors, any risks must have been considered absent or minimal, and evidence must be presented that the experiments were performed with the understanding and consent of the legal guardian.

All manuscripts must contain statements indicating that (i) informed consent has been obtained, preferably in writing, (ii) studies conformed to the standards set by the Declaration of Helsinki or the version that was in place at the time of the experiments, NOTE: If the research study was registered (clause 35 of the Declaration of Helsinki) the registration database should be indicated, else the lack of registration should be noted as an exception (e.g. The study conformed to the standards set by the Declaration of Helsinki, except for registration in a database.); and (iii) the procedures have been approved by the local ethics committee.

### DISCLAIMER

The Publisher, the Physiological Society and Editors cannot be held responsible for any errors in or any consequences arising from the use of information contained in this journal. The views and opinions expressed do not necessarily reflect those of the Publisher, the Physiological Society or Editors, neither does the publication of advertisements constitute any endorsement by the Publisher, the Physiological Society, Editors, or Authors of the products advertised.

Wiley Open Access articles posted to repositories or websites are without warranty from Wiley of any kind, either express or implied, including, but not limited to, warranties of merchantability, fitness for a particular purpose, or non-infringement. To the fullest extent permitted by law Wiley disclaims all liability for any loss or damage arising out of, or in connection with, the use of or inability to use the content.

Experimental Physiology

Vol 111.8

### Contents of volume 111.8

1 August 2026

### EDITORIAL

3445 An operational approach for estimating pulmonary diffusing capacity for oxygen in humans
*Stine B. Nymand, Rie Skovly Thomsen, Iben Elmerdahl Rasmussen and Ronan M. G. Berg*

3449 Artificial intelligence in contact sports research: Promise and peril for predicting and preventing brain injury
*Danny W. Walmsley and Damian M. Bailey*

3454 Why historians must learn from physiologists
*Katherine Hill and Lyndal Roper*

3458 Letters to the editor represent the apogee of peer review: A current exemplar
*Timothy I. Musch, Kiana M. Schulze and David C. Poole*

### VIEWPOINT

3461 When is a muscle at rest? The problem of background activity in brain stimulation
*Nicholas P. Holmes*

### METHODS AND TECHNIQUES

3463 Accuracy of next-generation wireless skin temperature sensors during exercise–heat stress
*Aaron J. E. Bach, Norman R. Morris, Surendran Sabapathy, David N. Borg, Isabella Ennever, Pramod Sharma and Fergus K. O'Connor*

3480 (Dis)agreement and concordance of metabolic indices from the oral glucose tolerance test and mixed-meal tolerance test: Implications for application
*Nina Sloth Nielsen, Nikolaj Agerlin, Nikolas Antoniou, Helga Ellingsgaard, Kristian Karstoft, Louise Lang Lehrskov, Grit Elster Legård, Mark Preben Printz Lyngbæk, Beckey Trinh, Thomas P. J. Solomon, Marc Y. Donath, Mathias Ried-Larsen and Cody Garett Durrer*

3492 Very low-amplitude muscle activity increases probability of motor evoked potentials in healthy individuals and in amyotrophic lateral sclerosis
*Séamus O'Sullivan, Yasmine Tadjine, Gabriel R. Palma, Narin Suleyman, Eva Woods, Antonio Fasano, Cathal Walsh, Friedemann Awiszus, Orla Hardiman, Richard G. Carson and Roisin McMackin*

### SHORT COMMUNICATION

3502 Impairments in mitochondrial dynamics and morphology in skeletal muscle of breast cancer patients after a single paclitaxel administration
*Joris Mallard, Elyse Hucteau, Laura Somme, Hervé Bischoff, Valérie Demais, Pauline Silvestrin, Anne-Laure Charles, Roland Schott, Xavier Pivot, Thomas J. Hureau and Allan F. Pagano*

### REVIEW ARTICLE

3514 The interplay between pulsatility, sympathetic regulation and renal physiology: Implications for left ventricular assist devices
*Tania Warnakulasuriya, Zaid Bahi and Rohit Ramchandra*

3525 Digital N-of-1 trials and their application in experimental physiology
*Stefan Konigorski, Mathias Ried-Larsen and Christopher H. Schmid*

3546 How physiology solves the gene-centric impasse
*Denis Noble and Reine Bourret*

### RESEARCH ARTICLE

### CARDIOVASCULAR CONTROL

3557 Therapeutic intervention with sinomenine and irisin to preserve cardiac function after ischaemia–reperfusion injury
*Lianjun Wu, Sining Bi, Amany Belal and Zhuzhu Liu*

3570 Apical hypercontractility mitigates impaired diastolic filling and lower intraventricular haemodynamic forces in human bed rest
*Jérémy Rabineau, Fatimah Al-Darwish, Edwin Mulder, Bram F. Coolen, Fabian Hoffmann, David Hautemann, Jens Tank, Pierre-François Migeotte, Gustav J. Strijkers and Rob C. I. Wüst*

3588 Dose-dependent effects of graded altered gravity during hypovolaemia on central haemodynamics and cardiovascular autonomic regulation
*Adrien Robin, Cort D. Reinarz, Huc Pentinat-Llurba, Syeda Yasmin Zaman, Christopher J. Rasmussen, Jason R. McKnight, Lisa Haddad, David C. Zawieja and Ana Diaz-Artiles*

3604 Haemodynamic responses to small muscle mass exercise before and after lung transplantation in end-stage chronic obstructive pulmonary disease
*Camilla Koch Ryrsø, Thomas Kromann Lund, Pia Thaning, Lasse Gliemann, Michael Perch, Jacob Peter Hartmann, Ida Kær Thorsen, Gregers Winding Munch, Stefan P. Mortensen, Ylva Hellsten, Ronan M. G. Berg and Ulrik Winning Iepsen*

### ENDOCRINOLOGY AND METABOLISM

3620 Independent and synergistic effects of maternal and paternal obesogenic diets on offspring early-life outcomes in the rat
*Khurram Jahangir Toor, Mehwish Abbasi, Swetha Muralidharan, Elisa Weiss, Elwyn C. Firth, Anna P. Ponnampalam, David S. Musson, Mark H. Vickers and Benjamin B. Albert*

3635 Substrate-specific mitochondrial dysfunction and metabolomic profiles in type 2 diabetic rat hearts
*Toan Pham*

3648 Acute capillary blood DNA methylation responses to swimming exercise in high-performance male and female swimmers
*C. D. Goldsmith, N. G. Lawler, D. B. Pyne, M. Kozlovskaia, K. McGibbon, L. J. G. Mitchell and A. D. Govus*

3667 Long-term adverse cardiovascular changes induced by cocoa shell extract administration through lactation in fetal undernourished and control rats
*Santiago Ruvira, Pilar Rodríguez-Rodríguez, Metee Iampanichakul, Begoña Quintana-Villamandos, Dolores Morales, David Ramiro-Cortijo and Silvia M. Arribas*

### ENVIRONMENTAL AND EXERCISE

3684 Effects of tumour necrosis factor and interleukin-6 inhibitor therapy on exercise-related visceral adipose tissue reduction in rheumatoid arthritis
*Simon Jønck, Malte Lund Adamsen, Iben E. Rasmussen, Anna A. Lytzen, Mathilde Løk, Morten Asp Vonsild Lund, Benjamin Nerup, Lene Wohlfahrt Dreyer, Peter G. Jørgensen, Niels Vejlstrup, Lars Køber, Søren Jacobsen, Bente Klarlund Pedersen, Helga Ellingsgaard, Pil Højgaard, Ronan M. G. Berg and Regitse Højgaard Christensen*

3699 Jumping in simulated lunar gravity with blood flow restriction as a potential exercise countermeasure: The acute physiological effects
*Patrick Swain, Filipa Santos, Nick Caplan, Cody Latue, Billie Illingworth, Nicky Sheard and Luke Hughes*

3718 Interindividual variability in static apnoea performance is partly explained by genetic factors
*Anastasios Makris, Alexandros Sotiridis, Nickos Geladas, Kalliopi Gkouskou, Vassilis Klissouras and Maria D. Koskolou*

### GI AND EPITHELIAL

3731 Timing-dependent protective and therapeutic effects of ethyl pyruvate in trinitrobenzene sulfonic acid-induced experimental colitis
*Hatice Yorulmaz, Elif Yorulmaz, Elif Özkök, Suat Hayri Küçük, Erdem Sünger, Ayyub Ebrahimi, Beyza Yılmaz and Serdar Altınay*

### HEART/CARDIAC MUSCLE

3742 Mitochondrial peptide MOTS-c suppresses systemic and cardiac inflammasome activation in a diabetic rat model
*Aimee R. Mills, Antonio de Souza, Toan Pham and Odunayo O. Mugisho*

### MUSCLE

3753 Phosphate and acidosis cause fibre type-specific changes to cellular and molecular contractile mechanics at 37°C in skeletal muscle from older adults
*Brent A. Momb, Jane A. Kent, Stuart R. Chipkin and Mark S. Miller*

3772 Circulating mitochondrial-derived microproteins at rest and in response to an acute bout of endurance exercise in individuals with cerebral palsy
*Oscar Horwath, Linnéa Corell, Junxiang Wan, Emma Hjalmarsson, Julia Starck, Stefan M. Reitzner, Jessica Norrbom, Rodrigo Fernandez-Gonzalo, Ola Kvist, Pinchas Cohen, Ferdinand von Walden and Sebastian Edman*

3784 Divergent skeletal muscle metabolite exchange in insulin-like growth factor-1-stimulated myotubes and resistance-exercised human muscle
*Tim Havers, Moritz Eggelbusch, Marius Meinhold, Kira Sing, Rainer Okrojek, Anna Artati, Michael Witting, Dominik Lutter, Claire V. Kieker, Collin Starke, Tushar More, Karsten Hiller, Flemming Dela, Martin Schönfelder, André Kafka, Stephan Geisler and Henning Wackerhage*

### NEUROSCIENCE

3800 Therapeutic potential of spexin in diabetic neuropathy: Insights from in vitro and in vivo models
*Mehmet Refik Bahar, Seval Ulku Orhan, Cigdem Tekin, Asli Taslidere, Mete Ozcan and Suat Tekin*

3818 Effects of baclofen and lorazepam on interhemispheric inhibition in humans
*Faith C. Adams, Karishma R. Ramdeo, Malaikah Ahmad, Stevie D. Foglia, Chloe C. Drapeau, Mustaali Hussain, Jiyeon Park, Mark A. Tarnopolsky and Aimee J. Nelson*

### RESPIRATORY

3828 The impact of statistical choice on variability and error in maximal respiratory mouth pressure testing
*Mitch Lomax, Josh Osofa, Nicola Armstrong, Michael Tipton and Gemma Milligan*

3843 Dendritic cell-specific hypoxia-inducible factor-1$\alpha$ deficiency protects mice from recurrent retroviral infection
*Timm Schreiber, Yvonne Hüsecken, Claudia Lodewick, Simone Schimmer, Gennadiy Zelinskyy, Ulf Dittmer, Kathrin Sutter, Joachim Fandrey and Sandra Winning*

### NOTIFICATION

3857 NOTIFICATION: Letters to the Editor Represent the Apogee of Peer Review: A Current Exemplar

Editor's Pick

O'Sullivan et al., demonstrate that very low amplitude muscle activity significantly increases the likelihood of evoking a muscle response with transcranial magnetic stimulation (TMS). This demonstrates that even minimal muscle activation can bias 'resting' TMS measures of motor excitability. See O'Sullivan et al. Pages 3492–3501.
